# How to improve information technology strategic planning effectiveness using balanced scorecard, risk and maturity analysis, case study health information technology? A qualitative study

**DOI:** 10.1002/hsr2.926

**Published:** 2022-11-18

**Authors:** Hossein Moinzad, Mohammad H. Akbarzadeh

**Affiliations:** ^1^ Department of Industrial Management Islamic Azad University Central Tehran Branch Tehran Iran; ^2^ Department of Industrial Engineering University of Science and Technology Tehran Iran

**Keywords:** BSC, COBIT, health information technology, maturity assessment, risk assessment

## Abstract

**Background and Aims:**

Although many health strategic plans have been developed by scholars and organizations, they still suffer from a limited view. Since most health‐related strategies in the future will depend on information technology (IT), as the main driver of today's industry, technology, and society, IT merits attention in health strategic plans. While the majority of the health strategic plan developed based on the interviews and questioner and these plans didn't consider the role of IT in their actions, this research will develop a framework to integrate risk and maturity analysis with the strategic planning process in health information technology strategic plan.

**Methods:**

The present research introduces an integrated framework based on a balanced scorecard (BSC) and control objectives for information and related technologies (COBIT). Also, The American Productivity & Quality Centre framework and COBIT were employed in this model to define the processes and activities of health IT (HIT) organizations. The organization's maturity and risk are analyzed in terms of information and management criteria using BSC, COBIT, and the analytical hierarchy process.

**Results:**

Later this model was implemented in a Remote Health care System to improve the strategic management process for this technology. Using this framework, 17 business goals have been developed and presented for the case. Also, the total related risk was 48% and the maturity level was at 1/18. The results are presented as decision‐making parameters and strategies for successful IT implementation.

**Conclusion:**

The presented framework provides deeper insight for the decision‐maker through integrating risk and maturity analysis in the HIT strategic planning process. The results are presented as decision‐making parameters and strategies for successful IT implementation. These strategies help investors decide about resource allocation based on the risk‐taking capability, certainty, and uncertainty results of the plan.

## 1 INTRODUCTION

Health information technology (HIT) is a combination of IT and all aspects of the healthcare domain, for example, medical sciences, health management, health economy, and nursing. All healthcare organizations and businesses should have a strategic plan for their future HIT based on visions, missions, and goals. Owing to the importance of HIT, considerable effort has been made to provide proper strategic plans in this field. The Office of the National Coordinator for HIT (ONC) is among the principal organizations developing strategic plans for HIT.^1^, ^2^ In addition to the ONC's strategic plans, various other plans^3^, ^4^, ^5^ have been developed and implemented for this purpose.

ONC presented its first HIT strategic plan in 2011 as the Federal Health Information Technology Strategic Plan,^1^ which commenced HIT implementation in the healthcare sector based on five goals, all aiming to achieve the acceptance and exchange of information, build trust among people in HIT, and empower individuals with HIT. Since this strategic plan was based on the opinion of experts and community information, it was a general and conceptual model that lacked computational accuracy and, therefore, could not communicate with nonfederal organizations. The strategies were not cost‐effective, and mobile‐based health apps were not employed.

The second plan of this office was developed and published in 2015 for 2015–2020.^2^ This strategic plan included four objectives and mainly sought to change personal and public health based on IT systems and improve the IT infrastructure. As a result, its implementation offers high‐quality care at a low cost and increases the healthy population. Nevertheless, its weaknesses include a lack of a global perspective for identifying and obtaining information from patients, the lack of harmonization of federal and state regulations, and the lack of risk analysis of the strategic plan and required measures for preventing risks.

Ritchie, William et al. indicated that in many hospitals and healthcare systems cost of doing business is the only tool to assess the quality of managers in this system. This study also indicated that the strategic goals of healthcare systems are achievable through the lens of balanced scorecard (BSC), and this tool could give new insights into the operational processes.^6^

In another study, the researcher shows that adopting BSC in the integration of big data and strategic management, not only will help to overcome complexities in the organizations, but also will help to improve the effectiveness of the strategic plan performance, and new insight will be available for managers decisions.^7^

In a study related to cloud adoption in healthcare systems, researchers found that the lack of interoperability and integration of the traditional healthcare information systems slowed down the expansion of e‐health. Although Cloud computing was a strategic goal for hospitals, there was complexity in execution due to low level of reliability and maturity in IT systems.^8^

The McKesson Corporation suggested that the strategic plan will be updated by developing roadmaps for high‐priority issues, encouraging collaboration on standard development, prioritizing administrative simplification, starting complete works to exchange the selected document types through direct exchange, harmonizing laws and regulations about health information privacy, security, and providing a risk‐based regulatory framework for HIT.^9^

In addition to the ONC strategic plans, various other plans^3^, ^4^, ^5^, ^10^, ^11^ were also developed and implemented in this field. Previous studies mainly have focused on providing strategic plans using methods such as surveys, questioners from experts and communities, and quality and cost analysis. However, since HIT is developing on IT infrastructures, it is necessary to analyze the risk and maturity of the HIT to prevent failures and casualties and increase the reliability of HIT systems. Whereas none of the previous studies were based on risk analysis and maturity assessment of the IT in the organization. Moreover, they did not address the quantitative criteria of IT, for example, reliability, resources, time, risk, and maturity, in the process of strategic planning. It is, therefore, necessary to analyze HIT risk and maturity since it is developed based on IT infrastructures. Hence by considering the importance of IT in the future of the Health sector especially by the emergence of the COVID‐19 pandemic, the lack of IT and health‐integrated planning framework to improve the effectiveness and efficiency of plans lead to the following research question: how can IT risk and maturity integrate into Health strategic planning to improve the effectiveness of the plan?

Thus, the main goal of this study is to provide a framework that aims to fill the research gap in previous plans and integrate risk and maturity assessment in the HIT planning process. The findings of risk and maturity analyses can serve as the input for developing a strategic plan for HIT technology execution and development.

In this paper, an integrated algorithm consisting of the balanced scorecard (BSC) model and COBIT framework is introduced for strategic plan development in HIT organizations. The strategic plan proposed for the HIT domain comprises risk and maturity analysis in terms of management metrics, which include the elevation of IT and its processes from four aspects of the BSC method.^12^

The COBIT framework was chosen because the procedures for the extraction of strategies from the strengths, weaknesses, opportunities, and threats (SWOT) analysis and the identification of KPIs from IT critical success factors (CSFs) are unknown in the BSC method.^13^ Moreover, IT projects are riddled with greater complexity due to real‐time and online integrated service‐oriented systems.

The algorithm proposed for strategic planning demonstrates the organizations' present maturity and risk ratio according to business goals (BGs) for each criterion. The proposed strategic planning model also presents the maturity and risk analysis for the health organization's current status to evaluate IT performance from each of the four BSC perspectives. The resulting strategic plan can offer an additional perspective to managers and decision‐makers to better understand HIT and prevent failures in HIT technology execution and expansion.

## 2 METHODOLOGY

**Figure 1:**
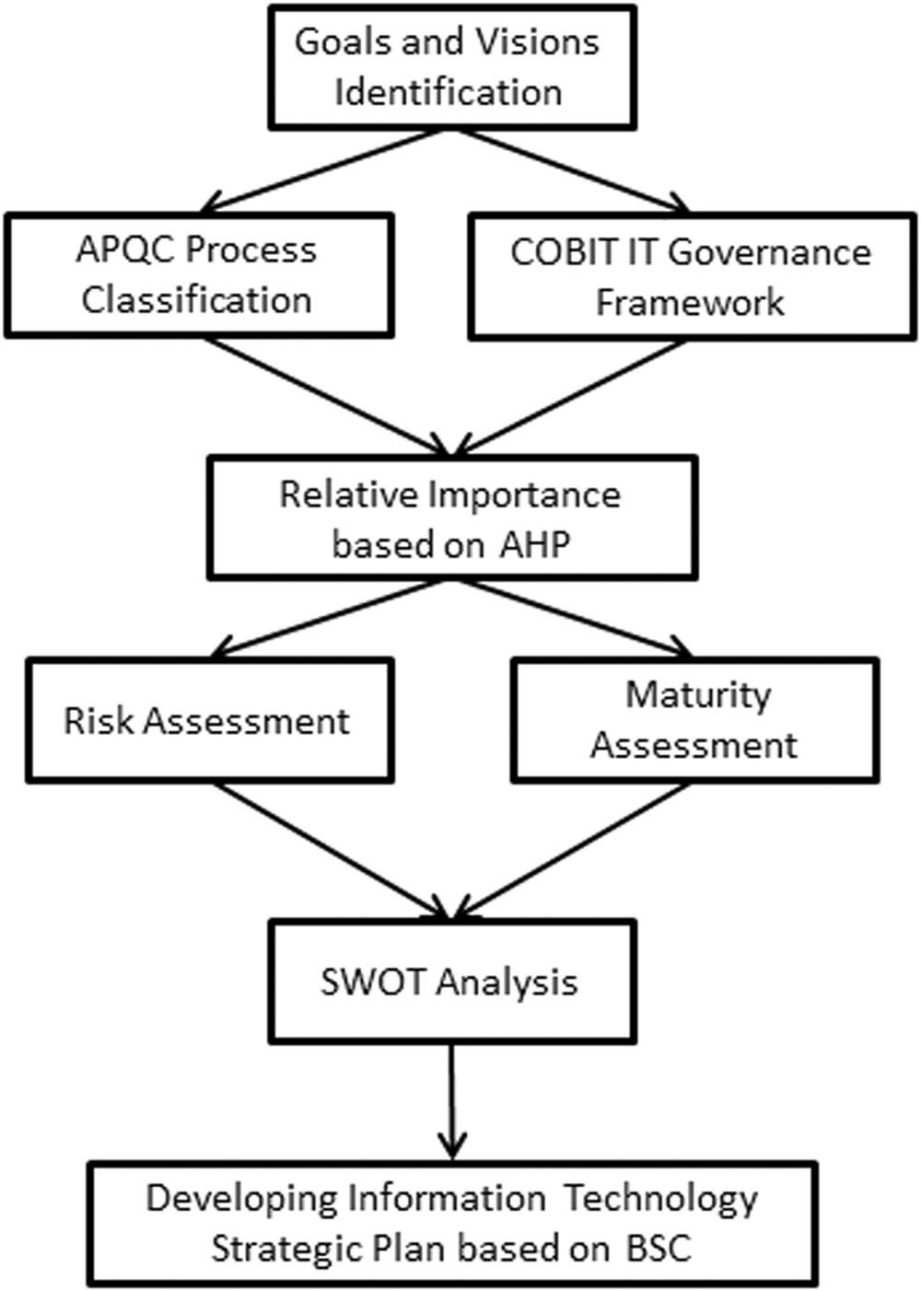
Control objectives for information and related technologies (COBIT)‐ and balanced scorecard (BSC)‐based strategic planning algorithm for successful health information technology (HIT) implementation

This study used focus group discussion as the method to gather data for SWOT analysis, identifying CSF's and developing the strategic plan. This paper proposed an eight‐step algorithm to develop a strategic plan for HIT implementation (Figure 1).

### 2.1 COBIT IT governance framework

**Figure 2:**
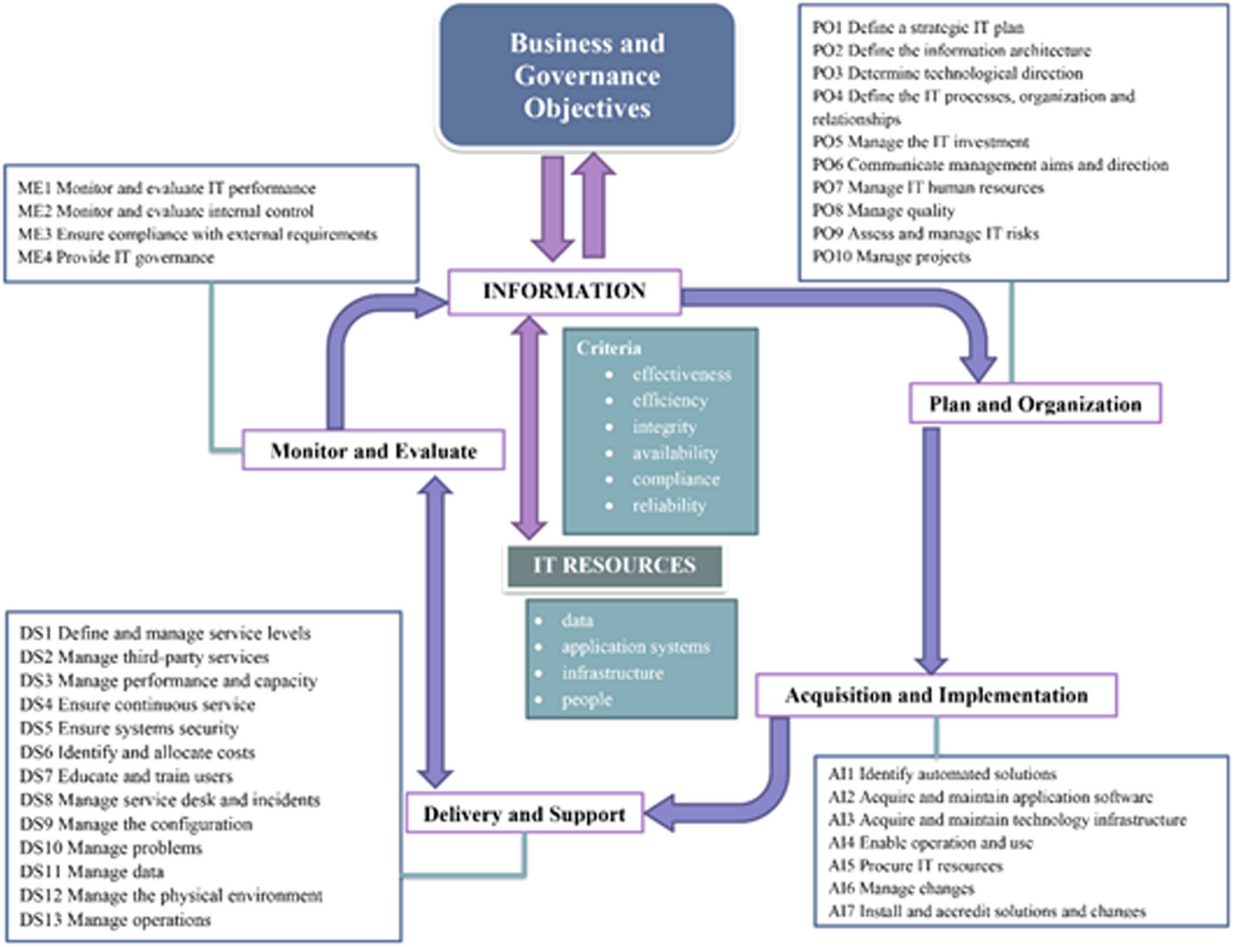
Control objectives for information and related technologies (COBIT) process framework^14^

Initially, four domains and 34 processes were defined according to the COBIT framework (Figure 2).^14^ The BGs and IT goals (ITGs) of COBIT were also reviewed and modified by experts for the organization.

### 2.2 APQC process classification

The APQC's industry‐specific process classification framework was adopted, and relative activities were identified.^15^ These activities were specified as 34 COBIT processes to form the hierarchical structure. A group of experts mapped the relations between APQC activities and COBIT processes to match and sort the APQC and COBIT framework results.

### 2.3 COBIT, BSC, and standard model

A comparison of the constituent elements of the COBIT 4.1, standard strategic planning, and BSC shows that:

- The actions common between the three systems include the development of strategic alignment visions, objectives, and performance evaluation.
- Both standard strategic planning and COBIT 4.1 obtain IT resource allocation and management and action plans.
- Both BSC and COBIT 4.1 insist on IT goals and BGs and strategic objectives' efficiency and effectiveness assessment.
- Standard strategic planning provides competitive advantages based on a strategic approach. Therefore, internal and external environment analysis and opportunity and threats analysis are required.
- COBIT 4.1 emphasizes risk analysis and maturity assessment of the organization's status quo.
- BSC focuses on the long‐term development of the organization and evaluates the strategic outcomes of the organization based on four perspectives.

**Figure 3:**
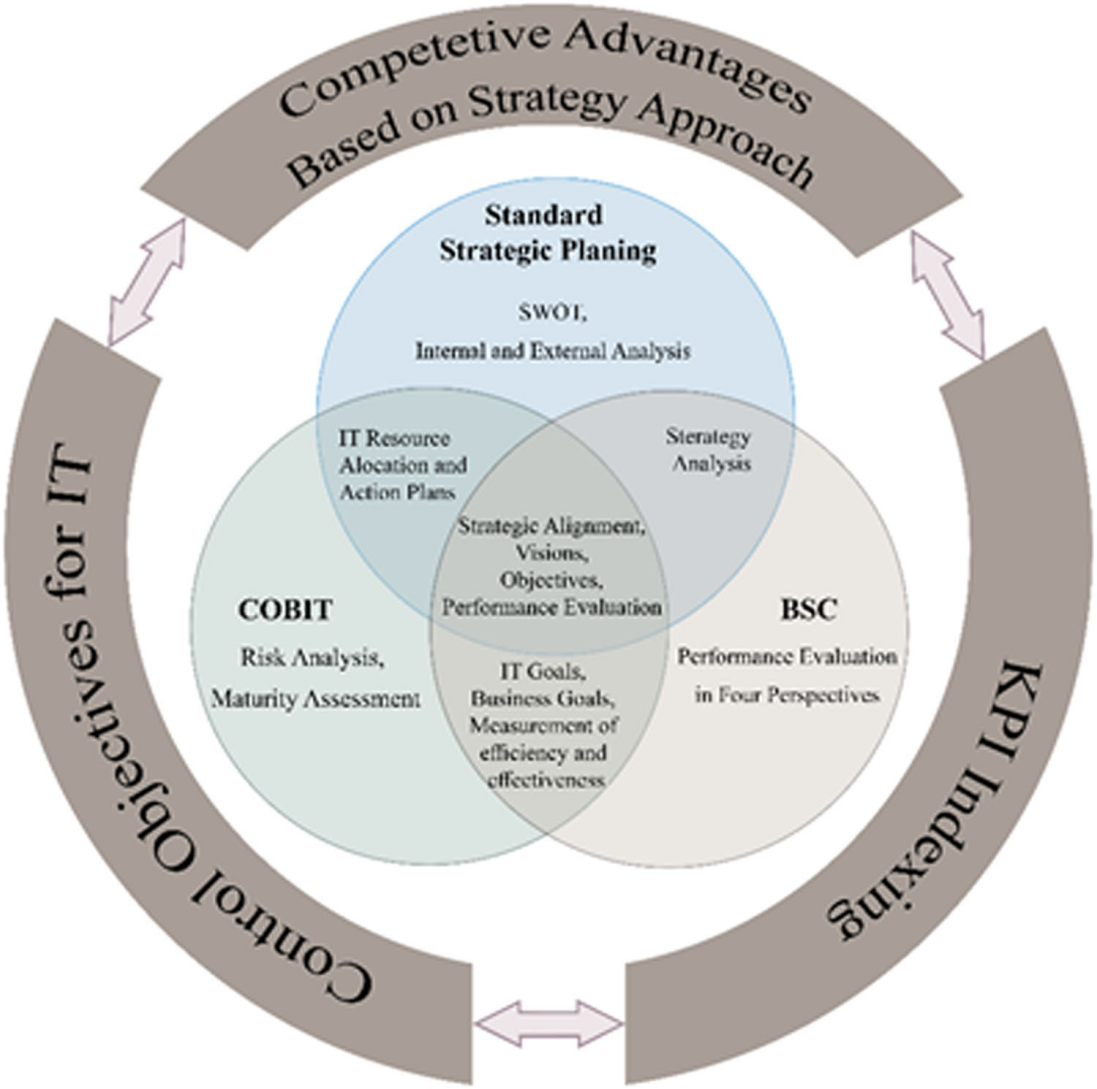
Comparison of control objectives for information and related technologies (COBIT), balanced scorecard (BSC), and standard strategic planning

A comparison of these methods is illustrated in Figure 3.

### 2.4 Relative importance (RI) based on AHP

In this step, time and cost were estimated for each IT‐related activity, and the RI of the activities of all processes was accordingly calculated. After that, based on the RI of activities and the relation map between activities and processes, the RI of each process in the organization was calculated by the AHP.^16^

### 2.5 Maturity assessment

Herein, maturity was calculated based on the COBIT 4.1 framework method. Based on this model proposed in the COBIT framework 11, organizations are classified into five levels of maturity, ranging from the initial to the optimized organization.

The only difference between the proposed and the COBIT methods is that COBIT assumes that all activities have the same weight and effect on the process maturity, whereas the proposed method assumed that the activities’ weight affects process maturity. A two‐step procedure was adopted to determine process maturity. In the first step, the process realization percentage was calculated based on the realization percentage of the related activities. In the second step, process maturity was determined by evaluating and calculating the number of attributes.

### 2.6 Risk assessment

To assess risk in the IT organization, 13 operational risks were first considered as the most common and risky incidents in IT projects, agreed upon by a team of experts:

(1) Inappropriate use of IT tools.
(2) Unstable published software.
(3) Integration of incorrect IT technologies with the existing infrastructure.
(4) Unrealistic expectations.
(5) Senior management's inadequate support.
(6) Insufficient attention to user requirements.
(7) Inadequate counseling.
(8) Higher‐than‐expected expenditure.
(9) Invalid IT management in the project.
(10) Excessive need for reliability.
(11) Knowledge management/invalid asset management.
(12) Security challenges.
(13) Low efficiency.

For risk calculation, the AHP model was employed in which the IT organization was placed at the top of the hierarchical model. The second level comprised four main domains: (1) planning and organizing, (2) receiving and implementing, (3) delivery and support, and (4) monitoring and evaluating. These four domains were converted into 34 processes at the third level. Next, the processes were turned into activities at the fourth level. At the fifth level, decision alternatives were placed, which included the 13 most common risks mentioned above.

**Figure 4:**
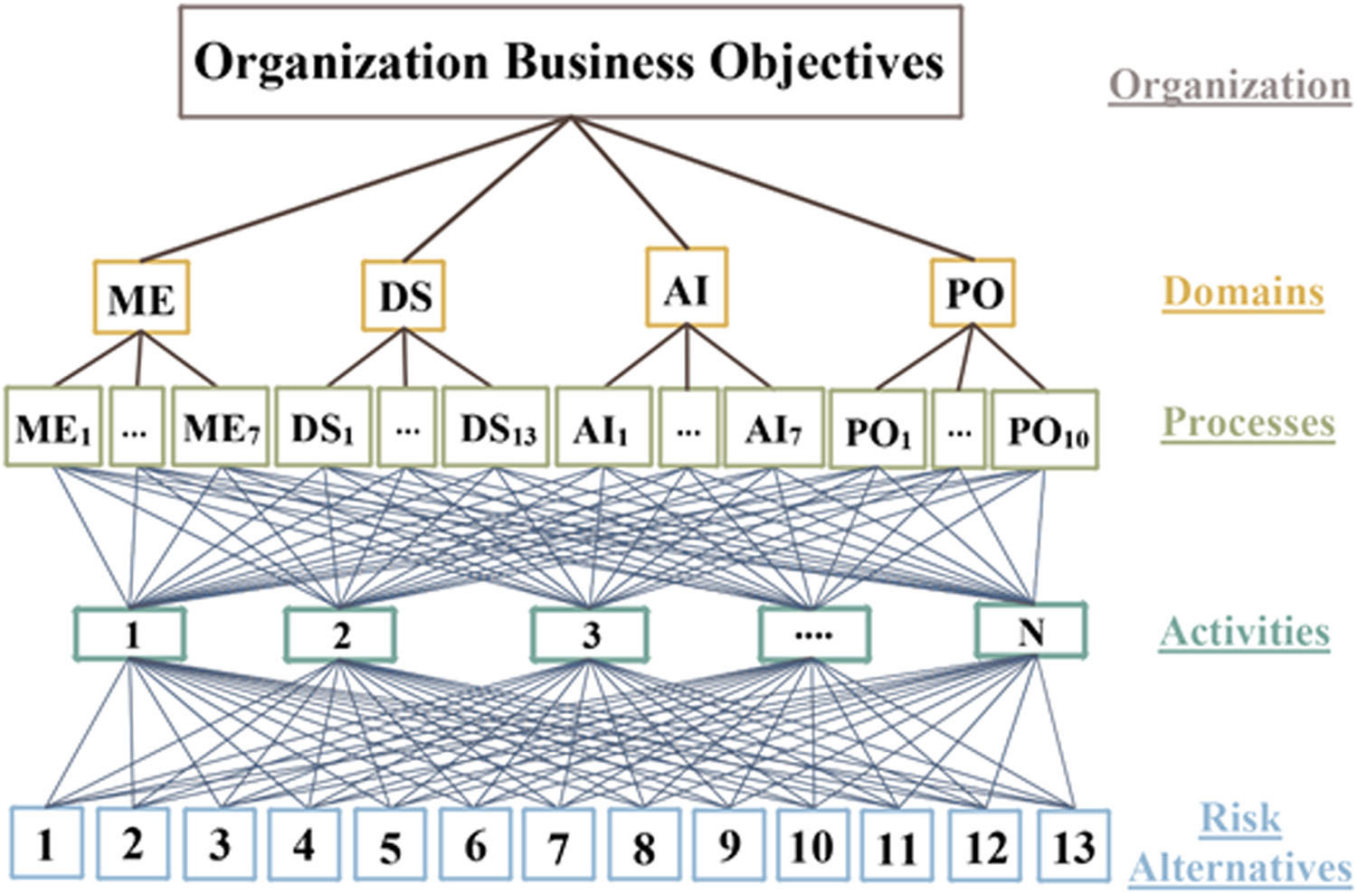
The implementation model of information technology risk assessment based on the analytical hierarchy process (AHP) process

Subsequently, risk analysis was conducted based on the AHP process, and maturity calculations were performed based on the COBIT framework. The RI for each process was also calculated from time, cost, and percent completed of activities. The hierarchy model is displayed in Figure 4.

### 2.7 SWOT analysis

In this step, the SWOT framework^17^ was utilized to evaluate the organization's competitive position and perform strategic planning.^18^ To determine the strengths and weaknesses, the maturity assessment results were used since they demonstrated the capabilities and flaws of the 34 processes and four domains of the IT organization. Next, the risk assessment results were applied to determine the opportunities and threats by providing valuable data on factors affecting the IT organization from external perspectives.

### 2.8 Developing the IT strategic plan based on BSC

The findings of the SWOT analysis were used by the group of experts, and strategies were developed for the related technology. The BGs resulting from SWOT was later reviewed by the experts and categorized under the four BSC perspectives.^12^ The strategies and data gathered from previous steps and the BSC were then employed to develop a strategic plan according to the related technology's risk and maturity.

Moreover, the risk and maturity of BGs and four BSC perspectives were calculated. according to the relations between 34 processes and ITGs, ITGs to BGs, and the RI. Knowing the maturity and risk of each perspective opens up horizons to managers and stakeholders for making more effective decisions.

## 3 IMPLEMENTATION

**Figure 5:**
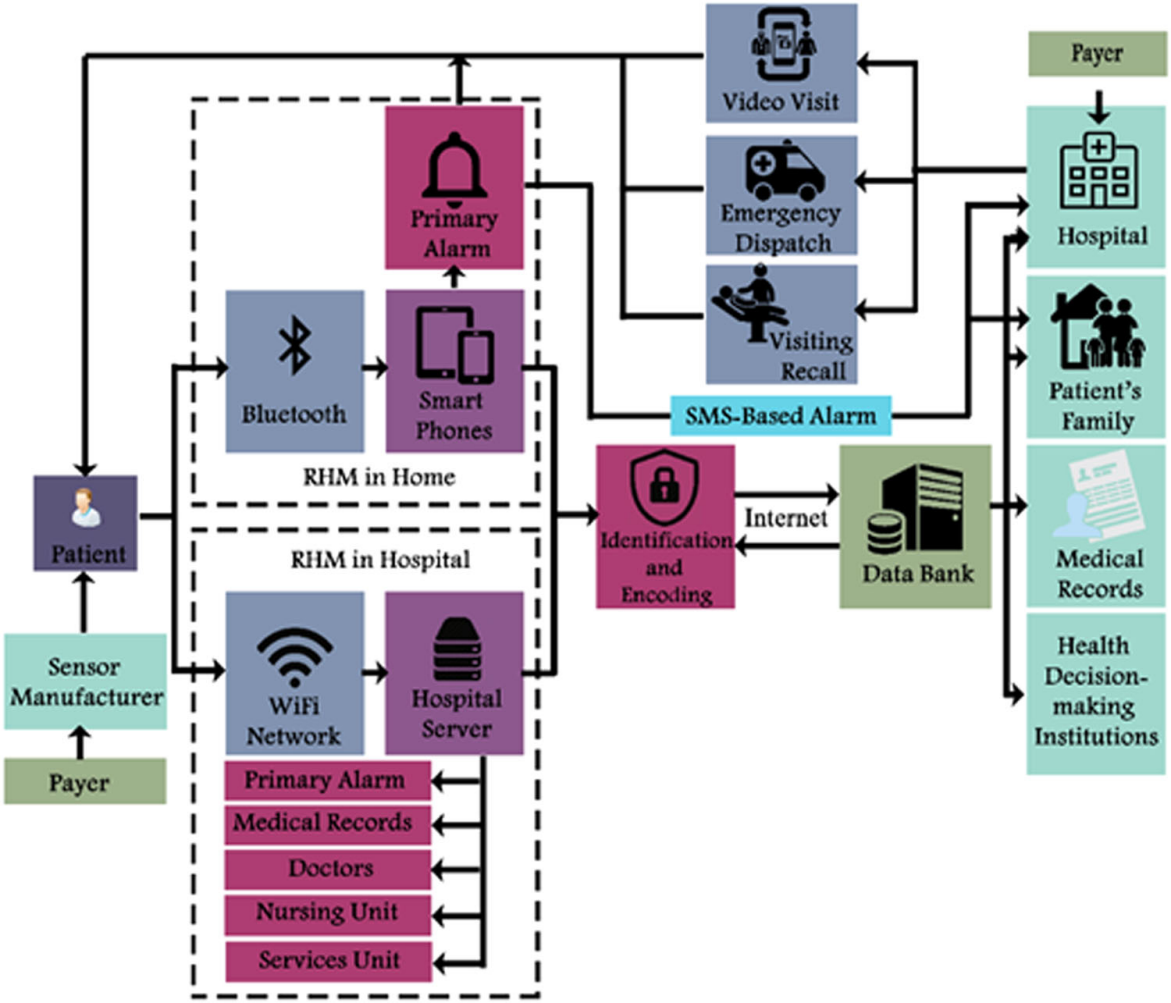
The conceptual architecture of the remote health monitoring (RHM) system

The remote health monitoring system (RHM) as a HIT technologywas adopted as the case study. RHMs are online multi‐dimensional decision‐making patient‐based health information processing networks proposed as a comprehensive tool for effective and efficient medical information sharing. The conceptual IT architecture of RHM is demonstrated in Figure 5.

Initially, a committee was assembled comprising 12 experts in healthcare, IT managers, and decision‐makers. The experts then reviewed and modified 28 ITGs of COBIT and adapted them to the case study. Consequently, 18 ITGs were identified for the HIT. In addition, the relationship between these 18 ITGs and the 34 processes of the COBIT framework was established based on the opinion of experts and organization managers. Table 1 lists the results.

**Table 1:** Eighteen information technology goals (ITGs) Based on control objectives for information and related technologies (COBIT) adapted to the case study

No	ITG	Related IT processes
1	Align the IT strategy to the business strategy.	PO1, PO2, PO4, PO10, AI1, AI6, AI7, DS1, DS3, ME1
2	Maintain the security (confidentiality, integrity, and availability) of information and processing infrastructure.	PO6, DS5, DS11, DS12, AI7, AI6
3	Make sure that IT services are reliable and secure.	PO6, PO8, AI4, AI6, DS1, DS2, DS4, DS7, DS8, DS10, DS12, DS13
4	Provide service offerings and service levels in line with business requirements.	PO8, AI4, AI6, AI7, DS10
5	Ensure IT compliance with laws and regulations.	PO1, PO4, PO10, ME1, ME2, ME3, ME4, DS11
6	Translate business functional and control requirements into effective and efficient automated solutions.	AI1, AI2, AI6
7	Deliver projects on time and budget, meeting quality standards.	PO8, PO10, DS2
8	Drive commitment and support of executive management.	PO9, DS10, ME2
9	Improve cost‐efficiency of IT.	PO5, DS6
10	Account for and protect all IT assets.	PO9, DS5, DS9, DS12, ME2
11	Acquire, develop and maintain IT skills that respond to the IT strategy.	PO7, AI5
12	Provide IT agility (in responding to changing business needs).	PO2, PO4, PO7, AI3
13	Offer transparency and understanding of IT costs, benefits and risks.	PO5, PO6, PO9, DS1, DS2, DS6, ME1, ME4
14	Optimize IT infrastructure, resources, and capabilities.	PO2, PO3, PO6, AI7, AI3, DS3, DS4, DS5, DS7, DS9, DS11, DS12, DS13, ME2
15	Accomplish proper use of applications, information, and technology solutions.	PO5, PO6, DS1, DS2, DS6, ME1, ME4
16	Seamlessly integrate applications and technology solutions into business processes.	PO2, PO3, AI2, AI3, AI4, AI5, AI7
17	Ensure that IT demonstrates continuous improvement and readiness for future change.	PO5, DS6, ME1, ME4
18	Acquire knowledge and expertise in emerging technologies for business innovation and optimization.	DS3, DS4, DS8, DS13

Following the proposed procedure, APQC introduced 13 main processes for healthcare service providers, which were then divided into 1985 activities. The experts adapted the APQC process framework to the case study. The committee developed a process map and refined these 1985 activities into 1634 activities in the case.

The activities were then categorized under the 34 processes of the COBIT architecture. Therefore, the RHM technology was divided into 1634 activities under 34 processes of the COBIT 4.1 framework. Afterward, the weight of each activity was calculated and was used to determine the RI of each process. Results are presented in Table 2.

**Table 2:** Relative importance (RI) in four domains and 34 processes

		Process RI in domain
Plan and organization	Domain RI	**29.20%**
	PO1 Define a strategic IT plan	10.8
	PO2 Define the information architecture	11.8
	PO3 Determine the technological direction	9
	PO4 Define the IT processes, organization, and relationships	9.5
	PO5 Manage the IT investment	9.3
	PO6 Communicate management aims and direction	8.5
	PO7 Manage IT human resources	9.5
	PO8 Manage quality	9.7
	PO9 Assess and manage IT risks	11
	PO10 Manage projects	10.9
Acquisition and implementation	Domain RI	**23.40%**
	AI1 Identify automated solutions	21.1
	AI2 Acquire and maintain application software	12.1
	AI3 Acquire and maintain technology infrastructure	10.8
	AI4 Enable operation and use	15.6
	AI5 Procure IT resources	14.3
	AI6 Manage changes	12.3
	AI7 Install and accredit solutions and changes	12.7
Delivery and support	Domain RI	**36.20%**
	DS1 Define and manage service levels	7.2
	DS2 Manage third‐party services	8.5
	DS3 Manage performance and capacity	7.9
	DS4 Ensure continuous service	7.8
	DS5 Ensure systems security	7.7
	DS6 Identify and allocate costs	7.3
	DS7 Educate and train users	7
	DS8 Manage service desk and incidents	7.6
	DS9 Manage the configuration	6.8
	DS10 Manage problems	7.6
	DS11 Manage data	10.2
	DS12 Manage the physical environment	7.1
	DS13 Manage operations	7.1
Monitor and evaluation	Domain RI	**11.20%**
	ME1 Monitor and evaluate IT performance	24.8
	ME2 Monitor and evaluate internal control	25.3
	ME3 Ensure compliance with external requirements	23.9
	ME4 Provide IT governance	26.1

Maturity was also calculated using the COBIT framework, and risk assessment was performed based on AHP (Table 3).

**Table 3:** Risk and maturity assessment results

Maturity level		Gained maturity	Process RI in domain	Risk level	Not achieved	Initial	Repeatable	Defined	Quantitatively managed	Optimizing
Plan and organization	Collected processes				Level 1/2					
	PO1 Define a strategic IT plan	2	10.8	52.2						
	PO2 Define the information architecture	1	11.8	59.6						
	PO3 Determine technological direction	1	9	57.1						
	PO4 Define the IT processes, organization, and relationships	1	9.5	58.5						
	PO5 Manage the IT investment	1	9.3	55.7						
	PO6 Communicate management aims and direction	1	8.5	56.6						
	PO7 Manage IT human resources	1	9.5	52.3						
	PO8 Manage quality	2	9.7	49.9						
	PO9 Assess and manage IT risks	1	11	46.4						
	PO10 Manage projects	1	10.9	49						
Acquisition and implementation	Collected processes				Level 1/11					
	AI1 Identify automated solutions	1	21.1	46.5						
	AI2 Acquire and maintain application software	1	12.1	47.4						
	AI3 Acquire and maintain technology infrastructure	2	10.8	47.2						
	AI4 Enable operation and use	1	15.6	45.1						
	AI5 Procure IT resources	1	14.3	47.7						
	AI6 Manage changes	1	12.3	42.4						
	AI7 Install and accredit solutions and changes	1	12.7	43.7						
Delivery and support	Collected processes				Level 1/08					
	DS1 Define and manage service levels	1	7.2	44.3						
	DS2 Manage third‐party services	1	8.5	45						
	DS3 Manage performance and capacity	1	7.9	42.1						
	DS4 Ensure continuous service	1	7.8	45.5						
	DS5 Ensure systems security	1	7.7	43.6						
	DS6 Identify and allocate costs	1	7.3	45.2						
	DS7 Educate and train users	1	7	44						
	DS8 Manage service desk and incidents	1	7.6	46						
	DS9 Manage the configuration	1	6.8	45.8						
	DS10 Manage problems	2	7.6	46.1						
	DS11 Manage data	1	10.2	49.6						
	DS12 Manage the physical environment	1	7.1	47.9						
	DS13 Manage operations	1	7.1	44.5						
Monitor and evaluation	Collected processes				Level 1/08					
	ME1 Monitor and evaluate IT performance	1	24.8	43.8						
	ME2 Monitor and evaluate internal control	1	25.3	47.5						
	ME3 Ensure compliance with external requirements	1	23.9	46.5						
	ME4 Provide IT governance.	1	26.1	45.4						

A SWOT analysis was conducted to analyze the advantages and disadvantages of the implementation of remote healthcare system (Table 4).

**Table 4:** Strengths, weaknesses, opportunities, and threats (SWOT) of health information technology (HIT)

**Strength**	
S1	Remote and immediate access to the clinical information of patients
S2	Security of patient information
S3	significant cost savings and operational benefits to the traditional healthcare system
S4	Improved patient safety
S5	Greater efficiency of operation
**Weaknesses**	
W1	Unreliability of communication channels
W2	The low business R&D budget
W3	Lack of efficiency assessment of investment
W4	low adoption of common legal/regulatory standards relevant for e‐Health
W5	Lack of system integration
W6	User resistance
W7	The slow trend of HIT Adoption
**Opportunities**	
O1	Aging population
O2	Health, demographic change, and wellbeing of society
O3	Availability of broadband connections
O4	Good investments in e‐Health
**Threats**	
T1	Rapid technological changes
T2	Lack of Policies/strategies in place for support of e‐Health services
T3	Low social willingness to accept e‐Health innovations
T4	Low cooperation of government and private sector
T5	Loss of patient trust
T6	Legal compliance

After that, the strategies extracted from the SWOT analysis were adopted, and the BSC was developed for the RHM (Table 5).

**Table 5:** Business goals extracted from strengths, weaknesses, opportunities, and threats (SWOT)

No	Business goals	Strategy type	S	W	O	T
1	Improve customer orientation and service.	WT		W1, W7		T2, T3, T5, T6
2	Ensure compliance with external laws and regulations.	ST	S3, S4			T2, T4, T5, T6
3	Establish service continuity and availability.	SO	S1, S3, S5		O2, O3, O4	
4	Manage (IT‐related) business risks.	WT		W1, W4, W5		T1, T5, T6
5	Offer competitive products and services.	WO		W2, W4, W6, W7	O1, O2	
6	Improve and maintain business process functionality.	SO	S1, S5		O3, O4	
7	Provide a good return on (IT‐enabled) business investments.	SO	S3, S5		O3, O4	
8	Acquire, develop and maintain skilled and motivated people.	WT		W1, W3, W4, W5		T1, T2, T6
9	Create agility in responding to changing business requirements.	WO		W2, W5, W7		
10	Obtain reliable and useful information for strategic decision‐making.	WT		W1, W2, W6, W7		T1, T2, T4, T5
11	Achieve cost optimization of service delivery.	WT		W3, W7		T1, T2
12	Optimize business process costs.	WT		W3, W5, W7		T1, T3, T2
13	Enable and manage business change.	ST	S1, S2, S5			T1, T3, T4
14	Improve and maintain operational and staff productivity.	WT		W1, W3, W4, W5		T1, T2, T6
15	Improve financial transparency.	SO	S1, S2, S5		O3, O4	
16	Ensure compliance with internal policies.	ST	S2, S5			T4, T5, T6
17	Identify, enable and manage product and business innovation.	ST	S1, S3, S5			T1, T3, T4

At this stage, the correlation between BGs and ITGs was determined (Table 6).

**Table 6:** Information technology goals (ITGs), business goals (BGs), and balanced scorecard (BSC) perspectives correlation

BSC Perspective	No	BGs	ITGs
Financial (corporate) perspective	1	Ensure compliance with external laws and regulations.	ITG2, ITG3, ITG5, ITG14
	2	Manage (IT‐related) business risks.	ITG2, ITG3, ITG5, ITG8, ITG10, ITG13, ITG14
	3	Improve financial transparency.	ITG5, ITG13
	4	Provide a good return on (IT‐enabled) business investments.	ITG9
Customer perspective	5	Establish service continuity and availability.	ITG3, ITG4, ITG7, ITG18
	6	Improve customer orientation and service.	ITG3, ITG18
	7	Obtain reliable and useful information for strategic decision‐making.	ITG2, ITG5, ITG13, ITG14
	8	Create agility in responding to changing business requirements.	ITG1, ITG7, ITG12
	9	Offer competitive products and services.	ITG9, ITG12
	10	Achieve cost optimization of service delivery.	ITG7, ITG9, ITG16
Internal perspective	11	Improve and maintain business process functionality.	ITG6, ITG16
	12	Improve and maintain operational and staff productivity.	ITG15, ITG16
	13	Ensure compliance with internal policies.	ITG5, ITG15
	14	Optimize business process costs.	ITG9, ITG14, ITG15, ITG16
	15	Enable and manage business change.	ITG1, ITG6, ITG12, ITG16, ITG17
Learning and growth perspective	16	Identify, enable and manage product and business innovation.	ITG7, ITG12, ITG17
	17	Acquire, develop and maintain skilled and motivated people.	ITG11

The 17 identified BGs and their assortment under the four BSC perspectives served as a basis for strategic roadmap development (Figure 5). Finally, the risk and maturity of BSC perspectives and BGs are presented in Table 7.

**Table 7:** Maturity and risk assessment of balanced scorecard (BSC) perspectives and business goals

No	Business goals	BG relative importance (RI) **%**	BG maturity	BG risk (%)	BSC perspective	BSC RI	BSC maturity	BSC risk (%)
1	Ensure compliance with external laws and regulations	12.31	1.04	47.67	Financial perspective	0.29	1.09	47.87
2	Manage (IT‐related) business risks	13.52	1.06	47.64				
3	Improve financial transparency	2.68	1.17	49.16				
4	Provide a good return on (IT‐enabled) business investments	0.80	2.01	50.69				
5	Establish service continuity and availability	5.98	1.14	45.66	Customer perspective	0.33	1.2	48.25
6	Improve customer orientation and service	5.03	1.16	45.24				
7	Obtain reliable and useful information for strategic decision‐making	8.24	1.15	48.61				
8	Create agility in responding to changing business requirements	7.34	1.17	50.12				
9	Offer competitive products and services	2.51	1.47	53.34				
10	Achieve cost optimization of service delivery	3.44	1.34	48.56				
11	Improve and maintain business process functionality	4.43	1	48.52	Internal perspective	0.33	1.12	48.47
12	Improve and maintain operational and staff productivity	5.89	1.06	48.01				
13	Ensure compliance with internal policies	4.37	1.11	48.21				
14	Optimize business process costs	7.10	1.22	47.81				
15	Enable and manage business change	11.21	1.15	49.20				
16	Identify, enable and manage product and business innovation	4.25	1.38	50.70	Learning and growth perspective	0.05	1.31	50.41
17	Acquire, develop and maintain skilled and motivated people	0.91	1	49.03				

The implementation of RHM in the organization has an average maturity level of 1.18 and an average risk level of 48%. Based on the COBIT framework, this level of maturity and risk means that processes can be implemented and primary goals can be achieved.

## 4 RESULTS

To meet management goals such as communicating with the organization's requirements, utilizing a specific model, identifying key resources, and defining management control goals, the need to design a model whose main features are focusing on business goals, being process‐oriented, controlling, and measurable is necessary. Providing IT services Business requirements include business strategies, service planning, organizational planning, and technology planning, five strategies are offered in IT.

- A service strategy that includes demand management and IT management processes.
- A Design strategy includes information security management processes, service continuity management, and supplier management.
- The transfer strategy includes configuration management and change management services and knowledge management processes.
- Operational strategy, which includes operations management and management of problems and risks.
- Improvement strategies include improvement and control processes at the service delivery level.

Some of the actions that are proposed to improve management services are:

- Preparation and design of the regulations and a comprehensive model of governance and management of IT and implementation of the IT governance organization procedures.
- Developing Strategic, Financial, and Operational Strategies for Information Technology Organization.
- Developing a model of value creation of IT components in particular by the headquarters based on the organization's management style.
- Organizational Exploitation The deployment of information technology in the components of the organization, including macrostructure identification, determining the levels of IT responsibilities, allocating IT units and pillars to the levels of responsibility.
- Identifying and assessing the requirements for how information technology interacts.
- A description of the IT process model according to each level of responsibility, including the area of responsibility for policy and oversight, the area of planning responsibility and project management, and the area of responsibility for the management and implementation of information technology projects.
- Identify the model and framework for assessment, control, and management of information technology organization to ensure the setting of the IT governance framework, ensure the transfer of the value and benefits of information technology, ensure optimization of information technology risks, ensure optimization of resources and realization of the Profit.
- Evaluating and determining the level of an organization's information maturity and comparing with the patterns of daylight saving time.

**Figure 6:**
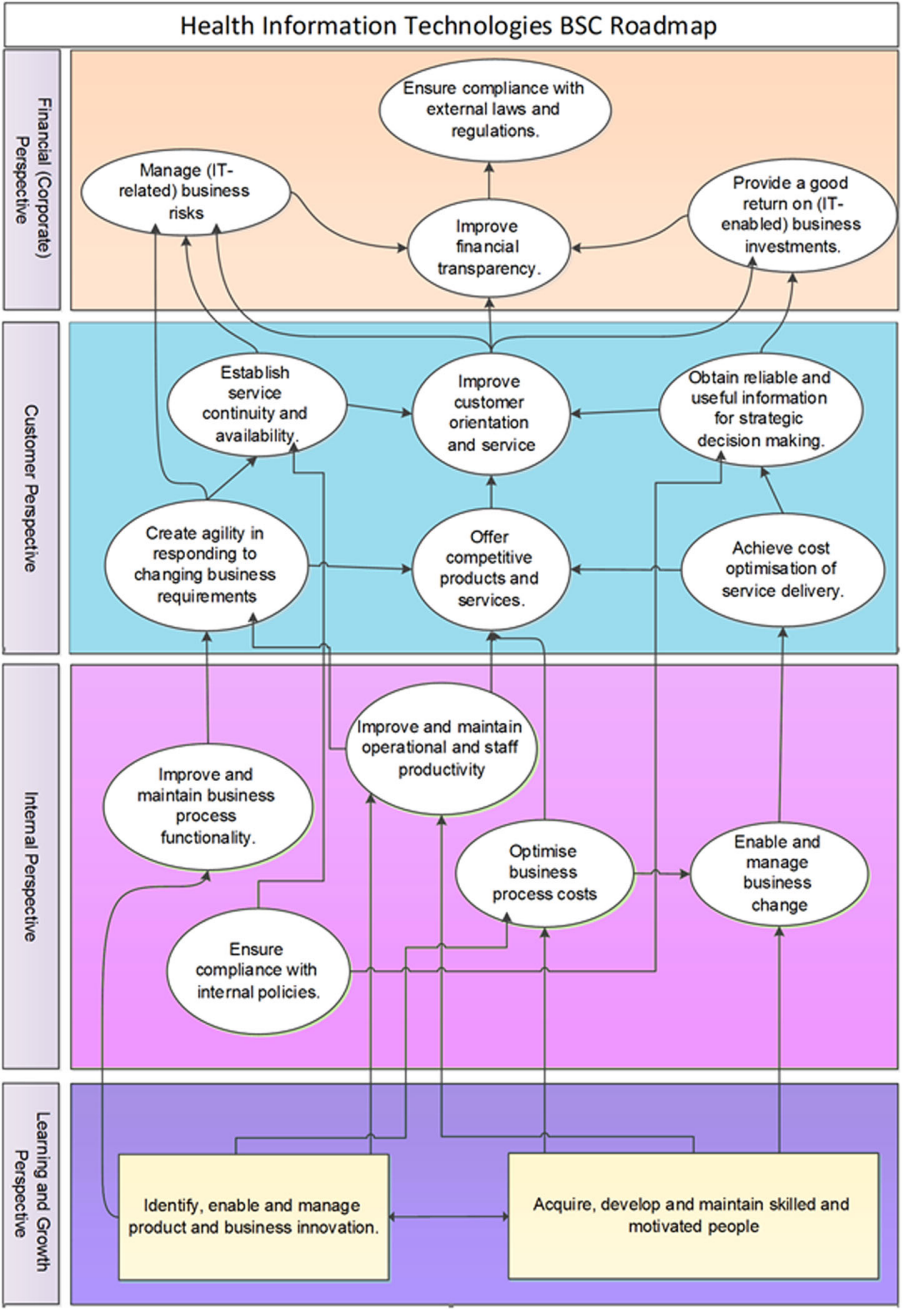
Balanced scorecard (BSC) strategic plan roadmap for health information technology (HIT)

The roadmap provided by this model can be seen in Figure 6, which is presented in four perspectives in accordance with BSC and contain 17 IT business goals of the HIT sector.

## 5 LIMITATIONS

The first limitation concerned the IT infrastructure. Since IT infrastructure reliability greatly impacts HIT risk and maturity, it could have affected the risk and maturity of the case study. The second limitation was related to the number of HIT experts (*n* = 12); although this number was sufficient for the analysis, it would be beneficial to increase it. The third limitation dealt with the case. This method was only used to develop a strategic plan for remote health care system; for its generalization, a strategic plan should be developed for other technologies.

## 6 CONCLUSION

Our goal with this paper was to show the importance of risk and maturity analysis in the HIT strategic planning process. Hence, we provided a comprehensive and integrative framework in which COBIT, AHP, and APQC models insights are used through balanced scorecard lenses to support strategy formulation and implementation. Throughout this paper, we emphasized that risk and maturity analysis insights could provide a deep and effective understanding for HIT strategy development. Traditional methodologies were based on SWOT analysis, questioners, interviews, and other strategic tools to provide clear environmental scanning. Not only, do risk and maturity analysis enhance the environmental scanning process, and provide accurate insights that help formulate better strategies, but also, they provide details to better understand future challenges in planning and made better decisions in advance. In terms of implementation, this study integrated the suggested framework with remote healthcare monitoring systems, which is one of the essential technologies in health systems in the future.

Herein, an integrated algorithm of BSC and COBIT was proposed and applied for the strategic planning of health and health‐related ICT. In this algorithm, activities were controlled through KPI, budget allocations, and performance evaluation systems to match organizational goals, ITGs, and business objectives to organizational missions and visions. It was concluded that, by assessing and analyzing risk and maturity in IT and developing a strategic plan, the performance of IT systems will be significantly enhanced, while the risk and cost of technology execution will be decreased (i.e., service quality, compatibility, support quality, information quality, usage time, accessibility, ease of use, response time, compatibility, social influence, understandability, and self‐efficacy). The IT management risk and maturity were evaluated by collecting data in 34 processes and four domains. The results of the paper can be applied to health‐related technologies to promote decision‐making for future strategies.

After calculating and confirming the results, the findings were provided to the organization's management to be exploited in:

(1) allocation of limited resources to activities and processes.
(2) Optimized and effective use of investment in IT.
(3) Administrative cost reduction.
(4) Effective use of IT for growth and development.
(5) Effective use of IT flexibility in commercial terms.

Future studies can utilize this algorithm and methodology for the strategic planning of other health technologies. Also, this framework could be used in other organization and industries which IT count as one of the most essential in developing strategic plans. In addition, future studies could be conducted with more experts in the field and using IT infrastructure reliability analysis in addition to risk and maturity analysis.

## AUTHOR CONTRIBUTIONS

**Hossein Moinzad**: Conceptualization; data curation; formal analysis; investigation; methodology; project administration; supervision; validation; writing – review and editing. **Mohammad H. Akbarzadeh**: Conceptualization; data curation; formal analysis; methodology; resources; visualization; writing – original draft; writing – review and editing.

## CONFLICT OF INTEREST

The authors declare no conflict of interest.

## TRANSPARENCY STATEMENT

The lead author Hossein Moinzad affirms that this manuscript is an honest, accurate, and transparent account of the study being reported; that no important aspects of the study have been omitted; and that any discrepancies from the study as planned (and, if relevant, registered) have been explained.

## Data Availability

The data that support the findings of this study are available on request from the corresponding author. The data are not publicly available due to privacy or ethical restrictions.

## References

[1] Office of the National Coordinator for Health Information Technology . *Federal Health Information Technology Strategic Plan 2011‐2015*. Office of the National Coordinator for Health Information Technology; 2011.

[2] Office of the National Coordinator for Health Information Technology . *Federal Health Information Technology Strategic Plan 2015‐2020*; 2015. Office of the National Coordinator for Health Information Technology.

[3] HHS USD of H& HS . *Strategic Plan Fiscal Years 2010–2015*; 2010.

[4] HHS USD of H& HS . *Strategic plan fiscal years 2014–2018*; 2014.

[5] HHS USD of H& HS . *Strategic Plan Fiscal Years 2018–2022*; 2018.

[6] Ritchie WJ , Ni J , Stark EM , Melnyk SA. *The Effectiveness of ISO 9001‐Based Healthcare Accreditation Surveyors and Standards on Hospital Performance Outcomes: A Balanced Scorecard Perspective*. https://www.tandfonline.com/doi/abs/

[7] Alnoukaria M . A framework for big data integration within the strategic management process based on a balanced scorecard methodology. J Intel Stud Bus. 2021;11(1):33‐47.

[8] Sharma M , Sehrawat R . A hybrid multi‐criteria decision‐making method for cloud adoption: evidence from the healthcare sector. Technol Soc. 2020;61:101258.

[9] Desalvo K , Ganley JM. *McKesson Comments on ONC's Federal Health IT Strategic Plan*; 2015. https://www.mckesson.com

[10] Koh HK , Berwick DM , Clancy CM , et al. *New Federal Policy Initiatives To Boost Health Literacy Can Help The Nation Move Beyond The Cycle Of Costly ‘Crisis Care’*; 2017. 10.1377/hlthaff20111169 PMC5102007 22262723

[11] Williams C , Mostashari F , Mertz K , Hogin E , Atwal P. *From The Office Of The National Coordinator: The Strategy For Advancing The Exchange Of Health Information*; 2017. 10.1377/hlthaff20111314 22392663

[12] Kaplan RS , Norton DP . Conceptual foundations of the balanced scorecard. Harvard Bus Rev. 2010;3:22. 10119714

[13] Yang CC , Yeh TM . An integrated implementation model of strategic planning, BSC and Hoshin management. Total Quality Management & Business Excellence. 2009;20(9):989‐1002.

[14] ISACA . *A Business Framework for the Governance and Management of Enterprise IT*; 2012.

[15] APQC.org . *APQC Process Classification Framework (PCF) – Healthcare Provider – Excel Version 6.1.1*; 2019.

[16] Saaty TL . Analytic Heirarchy Process Wiley StatsRef: Statistics Reference Online. John Wiley & Sons, Ltd; 2014.

[17] Hill T , Westbrook R . SWOT analysis: it's time for a product recall. Long Range Plan. 1997;30(1):46‐52.

[18] Alizadeh R , Soltanisehat L . Stay competitive in 2035: a scenario‐based method to foresight in the design and manufacturing industry. Foresight. 2020;22(3):309‐330.

